# Effectiveness of community outreach HIV prevention programs in Vietnam: a mixed methods evaluation

**DOI:** 10.1186/s12889-019-7418-5

**Published:** 2019-08-16

**Authors:** Lora L. Sabin, Katherine Semrau, Mary DeSilva, Loan T T Le, Jennifer J. Beard, Davidson H. Hamer, Jordan Tuchman, Theodore M. Hammett, Nafisa Halim, Manisha Reuben, Aldina Mesic, Taryn Vian

**Affiliations:** 1 Department of Global Health, Boston University School of Public Health, Boston, Massachusetts 02118 USA; 2 Department of Medicine, Harvard Medical School, Boston, MA USA; 3 Division of Global Health Equity, Brigham & Women’s Hospital, Boston, MA USA; 4 Ariadne Labs, Boston, MA USA; 5 University of New England, Portland, ME USA; 6 Ho Chi Minh City Statistics Office, Ho Chi Minh City, Vietnam; 7 Infectious Diseases Section, Department of Medicine, Boston University School of Medicine, Boston, MA USA; 8 United States Agency for International Development, Windhoek, Namibia; 9 Abt Associates, Cambridge, Cambridge, MA USA

**Keywords:** Mixed methods design, HIV prevention, Vietnam, Community outreach, Risk reduction behavior, Injection drug users, Sex workers, Men who have sex with men

## Abstract

**Background:**

In 2014, Vietnam was the first Southeast Asian country to commit to achieving the World Health Organization’s 90–90-90 global HIV targets (90% know their HIV status, 90% on sustained treatment, and 90% virally suppressed) by 2020. This pledge represented further confirmation of Vietnam’s efforts to respond to the HIV epidemic, one feature of which has been close collaboration with the U.S. President’s Emergency Plan for AIDS Relief (PEPFAR). Starting in 2004, PEPFAR supported community outreach programs targeting high-risk populations (people who inject drugs, men who have sex with men, and sex workers). To provide early evidence on program impact, in 2007–2008 we conducted a nationwide evaluation of PEPFAR-supported outreach programs in Vietnam. The evaluation focused on assessing program effect on HIV knowledge, high-risk behaviors, and HIV testing among high-risk populations—results relevant to Vietnam’s push to meet global HIV goals.

**Methods:**

We used a mixed-methods cross-sectional evaluation design. Data collection encompassed a quantitative survey of 2199 individuals, supplemented by 125 in-depth interviews. Participants were members of high-risk populations who reported recent contact with an outreach worker (intervention group) or no recent contact (comparison group). We assessed differences in HIV knowledge, risky behaviors, and HIV testing between groups, and between high-risk populations.

**Results:**

Intervention participants knew significantly more about transmission, prevention, and treatment than comparison participants. We found low levels of injection drug-use-related risk behaviors and little evidence of program impact on such behaviors. In contrast, a significantly smaller proportion of intervention than comparison participants reported risky sexual behaviors generally and within each high-risk population. Intervention participants were also more likely to have undergone HIV testing (76.1% vs. 47.0%, *p* < 0.0001) and to have received pre-test (78.0% vs. 33.7%, *p* < 0.0001) and post-test counseling (80.9% vs. 60.5%, *p* < 0.0001). Interviews supported evidence of high impact of outreach among all high-risk populations.

**Conclusions:**

Outreach programs appear to have reduced risky sexual behaviors and increased use of HIV testing services among high-risk populations in Vietnam. These programs can play a key role in reducing gaps in the HIV care cascade, achieving the global 90–90-90 goals, and creating an AIDS-free generation.

## Background

In Vietnam, HIV is concentrated among people who inject drugs (PWID), female sex workers (FSW), and men who have sex with men (MSM). Prevalence in the overall adult population has remained low, peaking in 2005 and remaining stable at 0.4% among adults aged 15–49 years [1]. By comparison, prevalence among PWID, FSW, and MSM is estimated to be 10.3, 2.6, and 3.7%, respectively [2], with PWID comprising 60% of all reported HIV-infected individuals [3]. Although these marginalized populations remain disproportionately affected, Vietnam has made impressive strides in reducing vulnerability and new infections since 2001, when prevalence peaked at 29.3% among male PWID and nearly 7.0% among FSW [4]. Data are limited for MSM, who have become a higher priority population in the last few years [5].

In 2014, Vietnam was the first Southeast Asian country to commit to achieving the 90-90-90 global HIV targets by 2020: 90% of population knowing HIV status, 90% on sustained antiretroviral therapy (ART), and 90% with suppressed viral load [6]. In June 2016 at the United Nations High Level General Assembly Meeting on Ending AIDS, Vietnam pledged to reach zero new infections by 2030 [6]. The United States government (USG) has invested in HIV prevention, testing, treatment, and care in Vietnam since the 1990s; in 2004, Vietnam was named one of fifteen focus countries by the President’s Emergency Plan for AIDS Relief (PEPFAR) [7]. Since then, PEPFAR investments have provided ART to 57,000 Vietnamese citizens, care and support to 62,000 children and adults, and methadone support for 25,000 PWID. In 2016, the USG committed $26 million through 2021 to help Vietnam meet the 90-90-90 targets. While support from other international sources, including direct bilateral aid from a number of governments (including Australia, Denmark, France, and the United Kingdom), United Nations organizations, and multilateral assistance (the Asian Development Bank, The Global Fund to Fight AIDS, Tuberculosis, and Malaria, and the World Bank) has played an important role in Vietnam’s HIV and AIDS achievements in meeting the 90-90-90 goals, PEPFAR has provided the greatest support. [8] According to the 2016 Country Operation Plan, Vietnam’s government has set an additional goal of independently funding 75% of the country’s HIV programming by 2020 [5].

In 2007–2008, we conducted a nationwide mixed-methods cross-sectional evaluation of the impact of PEPFAR-supported community-based outreach programs. The evaluation was designed to assess program effect on HIV knowledge, high-risk behaviors, and HIV testing among Vietnam’s key high-risk populations: PWID, FSW, and MSM. It also examined use of peer educators versus health educators, and made specific recommendations for program improvements. Initial findings were shared at a national workshop in Hanoi and in a USAID report [9, 10]; select descriptive results have been published elsewhere [11]. We focus here on the key findings related to the impact of the outreach programs on knowledge, risk-taking, and HIV testing uptake. Because this study was conducted 7 years before Vietnam committed to the 90–90-90 goals, it provides useful, detailed insight into the peer outreach efforts that have been central to government and development partner interventions.

While outreach efforts focused primarily on HIV testing and prevention, or what would later be articulated as the first 90 target, accompanying measures have been designed to increase capacity across all of the 90-90-90 goals. These capacity-strengthening measures include reducing stigma and discrimination, training physicians in clinical care and treatment of HIV-positive patients, developing a care and treatment coordination unit within the Ministry of Health, and establishing a national surveillance and monitoring system [12].

### Overview of Vietnam’s long-term peer outreach programming

Peer-driven community outreach efforts have been a core component of Vietnam’s domestic and international response to HIV. Vietnam first employed peer educators for HIV prevention among high-risk populations in 1993 [13]. In 2004, use of outreach workers was expanded in accordance with Vietnam’s National Strategic Plan on HIV/AIDS Prevention for 2004–2010, the country’s first HIV national plan [14]. With PEPFAR support, several internationally recognized best practices were adopted, including increasing voluntary HIV testing, implementing harm reduction measures for high-risk populations, and employing outreach workers to expand community-based HIV prevention measures focused on increasing HIV knowledge and reducing high-risk behaviors among key populations, primarily PWID, FSW, and MSM [15]. By 2007, PEPFAR-supported outreach programs had expanded in seven focus provinces (all high-prevalence provinces), and aimed to reach 30,000 individuals annually [16]. In the years since then, these programs have recruited, trained, and deployed cadres of peer educators (individuals who currently engage in injection drug use or sex work, or have in the past) and health educators (usually college graduates trained in health education). These outreach workers were trained to provide HIV-prevention information, skills, and supplies (such as condoms), as well as links to HIV testing, treatment, and other services, based on globally-recommended best practices for HIV prevention [12, 14].

Several studies have documented the impact of specific interventions based on use of peer outreach workers to engage with particular vulnerable populations in Vietnam. Hammett et al. (2012) described projects that have trained peer outreach workers to use personal education techniques and needle/syringe distribution to reduce HIV risk behaviors and infection among PWID in northern Vietnam and southern China [3]. In another study focused on adolescents and young adults aged 15–24 years, Ngo et al. (2013) described a combination of peer-employed education and integrated HIV and sexual and reproductive health services that successfully expanded HIV testing among youth [17]. A third study employed a pre-test, post-test design and found a positive association between engagement with peer educators and willingness to use pre-exposure prophylaxis among male sex workers in Ho Chi Minh City [18]. While these studies indicate the potential for specific interventions to make use of community outreach to contribute to desired behavior change, each was limited in scope to specific research questions, either focusing on one high-risk population or a single setting. None was designed to evaluate the broad impacts of community-based HIV prevention outreach programs operating across the country according to their original mission: to engage with all three of the major high-risk populations to increase their knowledge, reduce risky behaviors, and engage in HIV testing services.

While the data reported here were collected just over 10 years ago, the limited evidence published on this topic underscores the usefulness of this study for HIV researchers, program designers, and policy makers. To our knowledge, no other study in Vietnam has assessed the impact of community-based outreach programs at the national level among all of the primary high-risk populations and across multiple risk behaviors. Thus, these findings contribute vital evidence from which Vietnam and its development partners can build in the quest to reach the 90-90-90 targets by 2020 and achieve zero new infections, zero AIDS-related deaths, and zero stigma by 2030.

## Methods

### Design overview

Because outreach programs had been underway for several years, our design options were limited. To conduct an evaluation that could yield meaningful data on program impact, we used a mixed-methods, cross-sectional design that would permit comparison of relevant quantitative outcomes between an intervention group (individuals in each high-risk group with recent exposure to the outreach programs) and a comparison group (individuals in each high-risk group without recent exposure to the outreach programs). This was achieved through a comprehensive quantitative survey with members in each high-risk group. We supplemented the survey with qualitative in-depth interviews with survey participants to allow probing on important topics and triangulation of multiple sources of data. Our goal was to collect a rich set of quantitative and qualitative data to assess program impact related to knowledge and behaviors of populations known to engage in high-risk behaviors.

### Study sites and participants

Quantitative survey data were collected from members of high-risk populations in two northern provinces (Ha Noi, Hai Phong) and two southern provinces (Ho Chi Minh City (HCMC), An Giang). Qualitative data were gathered from individuals in the same populations in these four provinces and in two additional provinces (Quang Ninh in the north and Can Tho in the south). These provinces were selected because, due to their major HIV epidemics, they were focal provines of the PEPFAR-funded community outreach programs described above, and they provided geographic diversity across Vietnam. Ha Noi and HCMC are large, densely populated cities with modern economies, diverse populations from an influx of domestic migrants, and well-educated workforces. Hai Phong and Quang Ninh, on the northeastern coast, have relatively advanced infrastructure and large industrial workforces. Can Tho and An Giang are inland provinces with mainly agricultural economies and substantial migrant populations from neighboring Cambodia. All six provinces historically have had a relatively high HIV prevalence, particularly among PWID, for whom prevalence has recently ranged from 15 to 25% [19, 20].

Participants were recruited in collaboration with agencies implementing PEPFAR-funded outreach programs, including FHI 360 (formerly Family Health International), Ministry of Health/LIFEGAP, Médecins du Monde France, and Care International, using a snowball sampling approach. Peer outreach workers employed by the agencies made initial introductions to their regular clients, who in turn shared study information with their peers among PWID, FSW, and MSM. In accordance with our evaluation design, this included introductions to individuals who had had recent contact with an outreach worker in the previous 6 months and those who had not. These peers continued to introduce the study in subsequent rounds to their acquaintences until the desired sample size was achieved. Individuals were eligible if they were aged 18 years or older and could be categorized into one of four populations on the basis of self-reported behavior(s): 1) using injection drugs in the past month (PWID); 2) identifying as female, selling sex in the past month, and working on the street (SSW); 3) identifying as female, selling sex in the past month, and working in an establishment (KSW); or 4) identifying as a man who engaged in sex with another man in the past year (MSM). Participants who met multiple criteria were placed into a single primary group for recruitment purposes in this order: MSM, SSW/KSW, PWID.

The qualitative in-depth interviews (IDIs) were designed to collect supplemental in-depth information from survey participants about their personal experiences engaging with community outreach workers. Thus, in the four survey provinces, we randomly identified participants in the intervention group for IDI participation. In Quang Ninh and Can Tho (where no survey was conducted), we recruited IDI participants who reported recent contact with an outreach worker using the same snowball sampling approach described above.

All procedures performed in this study were in accordance with the relevant ethical standards of the institutional and national research committees and with the 1964 Helsinki declaration and its later amendments or comparable ethical standards. The study protocol was approved by the Institutional Review Boards of Boston University Medical Center, the Hanoi School of Public Health, and the HCMC AIDS Committee. All participants provided written informed consent.

### Data collection

Data were collected from November 2007 to September 2008, supervised by senior researchers at the HCMC Statistical Office. Trained supervisors from this office conducted workshops covering study purpose, methods, and ethical issues with the quantitative and qualitative research teams in each province. Field supervisors oversaw daily data collection. For the IDIs, six interviewers from Hanoi’s Institute of Sociology trained in qualitative methods collected data in Ha Noi, Hai Phong, Quang Ninh, and Can Tho. Six similarly trained interviewers from the Institute of Sociology in HCMC conducted IDIs in HCMC and An Giang. Two interviewers, one posing questions and the second taking notes, performed each IDI.

All surveys and IDIs were conducted face-to-face in Vietnamese in offices, club locales, or other locations convenient to participants. Each activity lasted 1.5–2 h. All participants received a stipend of 50,000 Vietnamese Dong (VND) (US$ 3.12) to cover the costs of travel and/or lost work time. In addition, participants who identified up to three other eligible survey participants were given an incentive stipend of 20,000 VND (US$ 1.25) per participant.

Survey questions were drawn from validated instruments previously used in Vietnam [21] that addressed HIV knowledge, health-risk behaviors, HIV testing, and interactions with outreach workers. Knowledge queries covered transmission (16 yes/no questions), prevention (17 yes/no questions), and treatment (8 yes/no questions). Health-risk behaviors included drug use, and needle sharing and cleaning; and protective behaviors related to condom access and use with primary, casual, and commercial partners in the previous 6 months. HIV testing questions asked about testing, as well as pre- and post-test counseling.

The IDIs employed semi-structured interview guides that allowed for open-ended responses and follow-up questioning. Questions covered program strengths and weaknesses, interactions with outreach workers, and perceived program impact. Survey and IDI instruments were pilot-tested and revised prior to data collection.

### Quantitative data analysis

High-risk populations and outreach programs were larger in Ha Noi and HCMC (“large provinces”) than in Hai Phong and An Giang (“small provinces”). In large provinces, we based sample size calculations on the ability to identify differences of 20 percentage-points or more in knowledge and high-risk behaviors between intervention and comparison groups for each population (using the most conservative estimate of 50% levels of correct knowledge for the comparison group), with a confidence interval of 95% (α = 0.05) and 80% power. In the small provinces, sample sizes for PWID, SSW, and KSW were half of those of the large provinces; because MSM programs were limited, we did not recruit MSM in small provinces.

Survey data were entered into Microsoft Visual FoxPro 9 by research staff in HCMC and analyzed in Boston using SAS version 9.0 (The SAS Institute, Cary, NC). We summarized responses using means, medians, and interquartile ranges for continuous variables; and frequencies and proportions for categorical variables. For knowledge questions, we constructed three sets of scores from the 16 yes/no transmission questions, 17 yes/no prevention questions, and 8 true/false treatment questions, respectively; each correct answer received one point. We calculated total proportion correct of the total possible score, and separate treatment and combined transmission/prevention scores, since the latter were the primary focus of outreach programs at the time of data collection. For sexual behavior questions, we defined consistent condom use as “always” (100%), based on the most conservative measure of condom use (anything below 100% use was considered inconsistent) [22].

In order to assess program impact related to HIV knowledge and behaviors, we disaggregated participants’ data into “intervention” vs. “comparison” groups based on whether they reported contact with an outreach worker in the most recent 6 months (intervention) or did not report such recent contact (comparison). We then compared intervention vs. comparison group differences in HIV/AIDS knowledge, high-risk behaviors, and HIV counseling and testing (HCT). We also compared responses among groups, with groups categorized by recruitment category (PWID, SSW, KSW, MSM) and by self-reported behaviors (injection-drug use, sex work, male-to-male sex). Because recruitment categories aimed to ensure an adequate number of participants within each of the population groups, whereas the behavior-based analysis encompassed all participants who reported a given behavior (allowing for overlap in behaviors when multiple behaviors were reported), we report findings based on self-reported behaviors as we believe they are more meaningful. We also report the results of analyses that stratify the FSW by SSW vs. KSW. We compared responses using χ^2^ tests for categorical variables and Student’s *t* tests for continuous variables.

### Qualitative data analysis

We aimed to conduct 6–10 IDIs with each of three high-risk populations (PWID, SSW/KSW, and MSM) in each province to assure a wide range of opinions. A professional translator translated audio-recordings into English; a Boston University Medical Center researcher checked each transcript and clarified unclear responses with the translator. Bilingual team members spot-checked transcripts for consistency with audio-recordings. One Boston-based analyst coded transcripts using a thematic approach and analyzed themes in Microsoft Excel 2010 [23]. We examined responses by population group and location. In addition to identifying common themes, we explored divergent views. We also identified illustrative statements to contextualize the results of the quantitative findings.

## Results

### Background characteristics of participants

We surveyed 2199 individuals, including 1100 intervention and 1099 comparison participants. Participants were generally young (mean age 29 years), evenly divided between male and female, and employed either full-or part-time (Table 1). Approximately one-fourth had no education beyond primary school. One-third reported “sex worker” as their main daily activity; others had a salaried job (14.3%) or performed manual labor (11.1%). Intervention participants were slightly older than comparison participants, and more likely to have tested positive for HIV (21.8% vs. 15.5%) and to have had sex with someone who is HIV-positive (13.1% vs. 5.7%) or a PWID (24.1% vs. 14.7%).

**Table 1:** Background Characteristics of High-Risk Population Survey Participants, By Intervention Vs. Comparison Group^a^

Characteristic	Intervention group (*N* = 1100)	Comparison group (*N* = 1099)
Recruitment category, number (%)^b^		
PWID	298 (27.1)	298 (27.1)
SSW	300 (27.2)	295 (26.8)
KSW	302 (27.5)	299 (27.2)
MSM	200 (18.2)	207 (18.8)
Location of survey, number (%)		
Hà Noi	400 (36.4)	408 (37.1)
Ho Chi Minh City (HCMC)	397 (36.1)	388 (35.3)
Hai Phòng	153 (13.9)	153 (13.9)
An Giang	150 (13.6)	150 (13.6)
Age in years, mean (SD)	29.6 (8.0)	28.5 (8.0)***
Gender (self-reported),^c^ number (%)		
Male	422 (38.4)	444 (40.4)
Female	646 (58.7)	629 (57.2)
Other	32 (2.9)	26 (2.4)
Education, highest level completed, number (%)		
Primary or none	267 (24.3)	311 (28.2)
Secondary (grade 6–9)	476 (43.3)	439 (39.1)
High school or higher	357 (32.4)	349 (31.7)
Employment, number (%)		
Full-time	529 (48.1)	509 (46.3)
Part-time	471 (42.8)	478 (43.5)
Unemployed	100 (9.1)	112 (10.2)
Mean monthly income (million VN dong)^d^ (SD)	2.911 (3.757)	2.672 (2.795)
Main daily activity, number (%)		
Construction/farming/petty job	221 (20.1)	224 (20.4)
Salaried job	139 (12.6)	177 (16.1)
Sex worker	394 (35.8)	352 (32.0)
Entertainment employee	106 (9.6)	89 (8.1)
Student, housework, other	141 (12.8)	146 (13.3)
Unemployed	99 (9.0)	111 (10.1)
Ever tested positive for HIV, number (%)	240 (21.8)	170 (15.5)**
Ever had sex with someone		
infected with HIV, number (%)	144 (13.1)	63 (5.7)***
Men who have sex with men	218 (19.8)	225 (20.5)
Individuals who inject drugs	265 (24.1)	162 (14.7)***

*SD* Standard deviation; **Significant at *p* < 0.01; ***Significant at *p* < 0.001

^a^Intervention group includes participants who reported contact with an outreach worker in the previous 6 months; comparison group includes those who reported no such contact

^b^Recruitment is based on self-reported: 1) using injection drug in the past month (PWID); 2) selling of sex in the past month and working on the street (SSW); 3) selling of sex and working in an establishment (KSW); 4) self-identifying as a male who engaged in sex with another man in the past year (MSM)

^c^Some participants declined to identify as either male or female

^d^The US$/VND exchange rate was approximately 16,280 at the time of data collection. In US$, reported mean incomes were approximately $178.8 (intervention) and $164.1 (comparison)

We conducted a total of 125 IDIs, 18–21 per province except in HCMC, where 25 IDIs were conducted (detailed data not shown). Qualitative participants’ characteristics were similar to those of survey participants: the mean age was 30.1 years; 48.0% were male; and one-fourth had no schooling above the primary level.

### Effectiveness of community outreach

#### Knowledge of HIV/AIDS

Overall knowledge of HIV transmission and prevention was high (mean score = 83.4%); treatment knowledge was poor (mean = 41.7) (Table 2). Across all knowledge categories, intervention participants scored significantly higher than comparison participants, particularly on treatment questions (48.2% vs. 35.2%, *p* < 0.0001). Within each separate key population group, intervention group scores were also significantly higher than comparison group scores for each knowledge category. Analysis by key population group found significantly higher scores among PWID on overall knowledge and on treatment, particularly compared to other key population groups; MSM scored highest on transmission/prevention knowledge. Among FSW, KSW participants scored higher than SSW in every category.

**Table 2:** Effectiveness of Community Outreach Programs: Knowledge of Key Populations, By Intervention vs. Comparison Groups

	All Topics (41 Questions)				Transmission & Prevention (33 Questions)				Treatment (8 Questions)			
	Overall	Intervention	Control	*P*-value	Overall	Intervention	Control	*P*-value	Overall	Intervention	Control	*P*-value
All participants n mean (SD)	2199 75.3 (11.6)	1100 78.2 (10.0)	1099 72.4 (12.4)	< 0.0001***	2199 83.4 (11.4)	1100 85.4 (9.3)	1099 81.5 (12.8)	< 0.0001***	2199 41.7 (27.3)	1100 48.2 (27.9)	1099 35.2 (25.0)	< 0.0001***
PWID n mean (SD)	694 76.4 (12.1)	358 79.1 (10.5)	336 73.5 (13.0)	< 0.0001***	694 83.6 (11.2)	358 85.4 (9.3)	336 81.6 (12.7)	< 0.0001***	694 46.7 (29.4)	358 53.2 (29.2)	336 39.8 (28.0)	< 0.0001***
MSM n mean (SD)	337 74.0 (10.2)	161 75.2 (9.3)	176 73.0 (10.9)	0.26	337 83.9 (10.0)	161 84.6 (8.6)	176 83.4 (11.1)	0.03*	337 33.0 (23.2)	161 36.2 (24.8)	176 30.1 (21.4)	< 0.0001***
All CSW n mean (SD)	1367 74.7 (12.0)	680 78.0 (10.3)	687 71.5 (12.7)	< 0.0001***	1367 82.9 (11.9)	680 85.2 (9.7)	687 80.6 (13.5)	< 0.0001***	1367 40.9 (26.8)	680 48.2 (27.5)	687 33.8 (24.0)	< 0.0001***
CSW Sub-Groups												
SSW n mean (SD)	594 74.3 (12.2)	300 77.6 (10.2)	294 71.0 (13.1)	< 0.0001***	594 82.7 (12.1)	300 84.8 (9.7)	294 80.5 (13.8)	< 0.0001***	594 39.9 (26.9)	300 47.9 (27.8)	294 31.7 (23.4)	< 0.0001***
KSW n mean (SD)	599 75.3 (12.1)	300 78.9 (10.3)	299 71.7 (12.6)	< 0.0001***	599 83.0 (12.3)	300 85.9 (9.8)	299 80.1 (13.7)	< 0.0001***	599 43.5 (26.8)	300 50.2 (27.2)	299 36.8 (24.8)	< 0.0001***

*SD* Standard Deviation; *Significant at *p* < 0.05; ***Significant at *p* < 0.001

Qualitative data from interviews underscored the ability of outreach workers to reach individuals at high risk of HIV infection with effective information about HIV transmission and ways to reduce HIV vulnerability. The majority of participants spoke of enhanced knowledge about HIV and risk reduction. Increased knowledge was also associated with behavior change, new attitudes about living with HIV, and reduced fear: *“I’ve changed … now that I know a little more, I’m not afraid anymore.”* (FSW in Ha Noi) More illustrative statements by participants are in Table 3.

**Table 3:** Illustrative Statements of HIV Risk Behaviors, Knowledge, Awareness, and Attitudes

Overall Knowledge, Awareness, and Attitudes Towards HIV	HIV Risk Behaviors	Counseling and Testing
*Overall Awareness* ▪ “I met her when I just started the job. I used to think nothing about disease, but after meeting [the outreach worker] I recognized the risk...I have to work, but I know how to avoid being infected by diseases.” – CSW in An Giang ▪ “We don’t learn much information from city wards or through papers. We learn more from [the outreach workers], just through normal conversation.” – MSM in Hà Noi *Transmission Routes* ▪ “At first when she gave me condoms, I asked her what they were for. She asked me if I [used] condoms. I said no. Then she said: ‘My goodness, you don’t use condoms, then you’ll catch sida [AIDS]’ At that time I didn’t know what sida [AIDS] was all about... I said: ‘You’re exaggerating that disease,’ I thought only injection drug users contracted that disease...[The outreach worker] gave me some books on HIV, I sat there and read, I understood. I was a bit scared.” – MSM in HCMC ▪ “Through [the HE], I know many more things. I am quite clear about the transmission of AIDS through sexual relations. Concerning diseases related to MSM sex, before I only knew some. When we talked, I asked a lot of MSM-related questions and she answered them.” – MSM in HCMC ▪ “I thought that [HIV] could be transmitted through sharing food or drink. Now I know that’s not true.” – CSW in Hà Noi ▪ “As she shows me ways of transmission, I feel more nervous … Since then, I use condoms with 100% of customers.” – CSW in An Giang *Attitudes Towards PLHIV, Being HIV+* ▪ “If I suffer from [HIV], I should lead an optimistic life. [I] shouldn’t be too pessimistic, and with hatred spread the disease out of revenge.” – PWID in Hà Noi ▪ “I used to think that if I get a positive [test] result, that’ll be the end of my life. Since I talked with the PE, I also want to have a blood test; so that I can have medicines to drink if I have disease.” –PWID in An Giang ▪“I’ve changed a lot. In the past, whenever I saw some dirty-looking sex workers, I dared not approach [them]. But now that I know a little more, I’m not afraid anymore.” – CSW in Hà Noi ▪ “I have learned and now know so much more … People living with HIV have to take regular blood tests and we should not discriminate against them. We should try to socialize with them and let them live and work.” – MSM in Can Tho	*Condom Use* ▪ “After talking [to the PE], I know how to protect myself. When I have sexual relations, I use condoms even with my lover. I’ve taught my boyfriend to use [condoms].” – CSW in Hai Phòng ▪ “When I was younger … sometimes 4 or 5 of us had sex with one girl and nobody used a condom. I was counseled and now I use condoms 100% [of the time].” – PWID in Hà Noi ▪ “Previously I didn’t use [condoms] much, but now I use condoms all the time. If customers don’t want to, I have to talk them into it. If customers still refuse to, I will have to walk out. Money is not everything.” – CSW in Can Tho ▪ “I changed my opinion about sex … I use condoms all the time I have anal sex.” – MSM outside of HCMC *Injection Drug Use* ▪ “I’ve changed my habits. I used to use a lot, so much that I had to steal from others. They [peers] advised me to use less... [Before we met the peer], we bought needles at drug stores to use...But I just rinsed them with boiling water, not with sterilizer like [the peers] show us.” – PWID in An Giang ▪ “Before meeting [outreach workers] I thought nothing about problems concerning sharing syringes and injection needles. Now, I no longer share syringes or needles with others.” – PWID in Hà Noi ▪ “In the past, when I was not aware, I had a libertine way of living...Now when they’ve told me, I try to avoid unsafe habits … [The PE] told me not to share needles.” – PWID in Hai Phòng ▪ Before having the chance to meet [the PE] and know the club, I used to share syringes and injection needles and had no knowledge of prevention. Since I met him, I have learned how to prevent HIV.” – PWID in Can Tho ▪ “When I didn’t know [the PE] … I still used old needles twice or three times... [Now] I apply the instructions she gives. For example, about using injection syringes and needles, I just use them for myself.” – PWID in An Giang ▪ “I’ve changed a lot, I’ve thought of it a lot … After listening to him, I don’t [share]. I used to use the kits again … Now I wash them with boiling water twice or three times according to the formula.” – PWID outside of HCMC	*Attitudes Towards Testing* ▪ “Why do I have to go for a test when I’m healthy? First, I think I’m healthy, and second, I don’t have the time … If someday I’m really weak, I’ll go there; now I don’t suffer from any disease, so I don’t go as it’s too far away.” – CSW in Hà Noi ▪ “I had the result after 1 week. Positive...I have no time; also, I felt less confident after that first test, so I won’t go there again. Maybe I’ll go to have a check again now. They have given me a letter to go to a medical establishment and get some medicine to take, but I haven’t gone. As I feel fine, I haven’t gone. I don’t go just because I don’t have time … [The PE] also advised me that just one time is not enough for an accurate result. I should have a check for one or two more times. But I don’t have time to go.” – PWID in Hai Phòng ▪ In response to: ‘Are the testing and counseling services helpful’? “Yes, [they are] very useful because these are the things that we need to know. Knowing a little is not the same as really knowing – MSM in Can Tho *Attitudes Towards Health Workers* ▪ “I totally believe in those places [HIV testing and counseling clinics]. The staff counseled me a lot and solved a lot of my problems. I could get a free test there, but I had to pay for the medicines.” – MSM in Hà Noi ▪ “The truth is, if they are in a good mood, they ask us questions; if not, they ask nothing … [When a staff person called out his name] I raised my objection, but they said it’s their duty. So I didn’t feel comfortable... Frankly speaking, we are drug addicts … we are still discriminated [against]; they look down on us. From my heart, I realize that.” – PWID in Hai Phòng ▪ “We can have HIV testing there and the outreach workers are very enthusiastic about providing us with counseling and advising us. When we receive our results, they also caution us to not overlook anything, and that … if I need to know anything, they will explain.” – MSM in Can Tho ▪ “The way they greeted us really offended us. [The receptionist’s] manner was very hierarchical, and she shouted and scolded us.” – CSW in Hà Noi

*PE* Peer educator, *CSW* Commercial sex worker, *PWID* People who inject drugs, *MSM* Men who have sex with men

#### High-risk injection behaviors

Among survey participants who reported using injection drugs in the previous 6 months (*n* = 694, 31.6%), we found low proportions of high-risk injection-related behaviors (Table 4). Only 14% reported having shared injection equipment in the previous 6 months; of those, 36% reported re-using a needle/syringe at last injection (data not shown). Few differences between intervention and comparison groups emerged. However, when asked about measures taken to reduce infection risk, a significantly higher proportion of intervention vs. comparison participants reported various actions, most notably, stopping sharing of injection equipment (74.6% vs. 67.9%, *p* = 0.05) and starting or increasing cleaning of equipment (62.2% vs. 52.1%, *p* < 0.001). A within-group analysis comparing PWID who were also SSW (*n* = 67), KSW (*n* = 23), or MSM (*n* = 8) vs. those who were only PWID (*n* = 596) showed that the latter were significantly more likely to have stopped sharing needles (59.1% vs. 34.8–46.3% for the overlapping groups, *p* = 0.029) or to have started/increased cleaning of equipment (59.7% vs. 25.0–47.8%, *p* = 0.009) (data not shown).

**Table 4:** Injection-related Behaviors by Intervention vs. Comparison Group, Among 694 People Who Inject Drugs

	Intervention (*n* = 358)		Comparison (*n* = 336)		*P*-value
When injecting in the last 6 months:	N	%	N	%	
Did not share needles/syringes or other injection equipment	306	85.5	288	85.7	0.90
Reduced injection of drugs	188	52.5	171	50.9	0.66
Stopped sharing equipment	267	74.6	228	67.9	0.05
Stopped sharing drug solutions	215	60.1	179	53.3	0.07
Started/increased cleaning works	223	62.2	175	52.1	< 0.01**
Always cleaned needles/syringes that had been previously used by someone else	14/46	30.4	8/46	17.4	0.11

**Significant at *p* < 0.01

Analysis of qualitative data (Table 3) found evidence supporting changed behaviors, particularly regarding sharing of syringes and needles and sterilization of equipment. Typical statements were: *“After meeting [the peer educator], I think I am more knowledgeable. If I want to inject, I have to do it carefully”* (PWID in Can Tho) and *“In the past, I thought: what would be, would be. I used drugs rashly until I was informed. I was also scared, [but] now I never share injection syringes and needles”* (PWID in Ha Noi).

#### High-risk sexual behavior: inconsistent condom use

Among the 2040 participants (92.8%) who reported having had vaginal or anal sex in the previous 6 months, large proportions reported high-risk sexual behaviors (see Table 5). Overall, only 37.5% reported “always” using a condom, while 16.7% “occasionally” used them (data not shown). We also found that a significantly higher proportion of intervention participants reported more frequent condom use, in general, and with each type of sexual partner—primary, casual, and client—in the previous 6 months. Among population groups, a higher proportion of FSW reported “always using” condoms (38.7%) compared to other groups; PWID reported the lowest consistent condom use (31.3%). Within each group, consistent condom use was highest when having sex with a client, with proportions ranging from a low of 42.7% (PWID) to a high of 63.8% (FSW). Additionally, the differences in consistent condom use between intervention and comparison participants were greatest with client sex partners for both FSW (69.9% v. 57.8%, *p* < 0.01) and PWID (61.5% v. 46.0%, *p* = 0.09); among MSM, however, this difference was highest for sex with casual partners (42.2% v. 32.3%, *p* = 0.01). MSM also reported similar levels of condom use across partner categories: approximately 40% reported “always use condoms” regardless of partner, whereas PWID and FSW reported much lower consistent condom use for casual partners (7–13%) and primary partners (24–28%) than for clients. Among FSW only, SSW tended to report somewhat higher consistent condom use than KSW, including with clients (67.8% v. 64.9%). Less than one-half (45.6%) of all participants reported “always carry condom,” with a significant difference between intervention and comparison participants (49.7% v. 41.4%, *p* < 0.01). This difference remained significant for FSW overall (55.9% v. 46.6%, *p* < 0.01), and for SSW (65.0% v. 55.2%, *p* = 0.02), whose reported “always carry condom” proportion was the highest of all groups.

**Table 5:** Risky Sexual Behaviors by Intervention vs. Comparison Group and Key Population

	Have ever used a condom n (%)				Among those who ever used a condom, always (100%) uses a condom n (%)				When having sex with primary partner in last 6 months, always (100%) used a condom n (%)				When having sex with casual partners in last 6 months, always (100%) used a condom n (%)				When having sex with client in last 6 months, always (100%) used a condom n (%)				Among condom users, always carries condoms n (%)			
	Overall	Control Intervention	Comparison	*P* Value	Overall	Intervention	Comparison	P Value	Overall	Intervention	Comparison	*P* Value	Overall	Intervention	Comparison	*P* Value	Overall	Intervention	Comparison	*P* Value	Overall	Intervention	Comparison l	*P* Value
ALL	2040/2199	1044/1100	996/1099	0.004**	765/2040	421/1044	344/996	0.001**	411/1403	245/737	166/666	0.0003***	316/2199	180/1100	136/1099	0.01*	873/1370	476/681	397/689	< 0.01**	931/2040	519/1044	412/996	< 0.01**
	(92.8)	(94.9)	(90.6)		(37.5)	(40.3)	(34.5)		(29.3)	(33.24)	(24.9)		(14.4)	(16.4)	(12.4)		(63.7)	(69.9)	(57.6)		(45.6)	(49.7)	(41.4)	
PWID (*n* = 694)	572/694	313/358	259/336	0.001**	179/572	108/313	71/259	0.05	88/368	57/205	31/163	0.04*	46/694	30/358	16/336	0.01*	71/128	48/78	23/50	0.09	195/572	115/313	80/259	0.16
	(82.4)	(87.4)	(77.1)		(31.3)	(34.5)	(27.4)		(23.9)	(27.8)	(19.0)		(6.6)	(8.4)	(4.8)		(55.5)	(61.5)	(46.0)		(34.1)	(36.7)	(30.9)	
MSM (*n* = 337)	312/337	149/161	163/176	0.98	115/312	60/149	55/163	0.16	94/263	50/125	44/138	0.32	125/337	68/161	57/176	0.01*	56/131	25/55	31/76	0.05	96/312	50/149	46/163	0.31
	(92.6)	(92.5)	(92.6)		(36.8)	(40.3)	(33.7)		(35.7)	(40.0)	(31.9)		(37.1)	(42.2)	(32.3)		(42.7)	(45.5)	(40.8)		(30.8)	(33.6)	(28.2)	
CSW (*n* = 1367)	1340/1367	672/680	668/687	0.03*	519/1340	281/672	238/668	0.01*	257/931	152/481	105/450	0.01*	171/1367	96/680	75/687	0.09	872/1367	475/680	397/687	< 0.01**	687/1340	376/672	311/668	< 0.01**
	(98.0)	(98.8)	(97.2)		(38.7)	(41.8)	(35.6)		(27.6)	(31.6)	(23.3)		(12.5)	(14.1)	(10.9)		(63.8)	(69.9)	(57.8)		(51.3)	(55.9)	(46.6)	
CSW sub-groups																								
SSW (*n* = 594)	588/594	300/300	288/294	0.01*	242/588	128/300	114/288	0.23	112/380	152/481	48/186	0.58	56/594	33/300	23/294	0.11	403/594	223/300	180/294	< 0.01 **	354/588	195/300	159/288	0.02*
	(98.9)	(100.0)	(98.0)		(41.1)	(42.6)	(39.6)		(29.5)	(31.6)	(25.8)		(9.4)	(11.0)	(7.8)		(67.8)	(74.3)	(61.2)		(60.2)	(65.0)	(55.2)	
KSW (*n* = 599)	592/599	299/300	293/299	0.05	231/582	128/299	103/293	0.01*	109/414	70/224	39/140	0.03*	63/599	36/300	27/299	0.18	389/599	211/300	178/299	0.01*	284/592	155/299	129/293	0.06
	(98.8)	(99.6)	(97.9)		(39.7)	(42.8)	(35.2)		(26.3)	(31.3)	(27.9)		(10.5)	(12.0)	(9.0)		(64.9)	(70.3)	(59.5)		(47.9)	(51.8)	(44.0)	

*Significant at *p* < 0.05; **Significant at *p* < 0.01; ***Significant at *p* < 0.001

Qualitative data (see Table 3) support these findings of effective messaging by outreach workers. Intervention participants described changing their attitudes and behaviors regarding condom use, with typical statements including, *“After talking to them [peer educators], I know a lot. After reading the papers they give, I changed my habits to be safer”* (FSW in Quang Ninh) and “*My partner has sex with others, so I use condoms all the time. Believe him or not, I still have to use condoms to be safe first*” (MSM in HCMC). Several participants revealed less than consistent condom use, however. One PWID in Ha Noi explained that sometimes late at night, when he was craving drugs, he would “*go out with friends and girls, and no one brings condoms.”*

#### HIV counseling and testing behaviors

More than half of respondents reported receiving pre-test counseling (55.8%) and having been tested for HIV (61.6%) (Table 6). Among those tested, 73.1% received post-test counseling and 89.9% received results. We found significant differences between intervention and comparison participants for each service except for receipt of results. The intervention group was significantly more likely to have been tested for HIV (76.1% vs. 47.0%, *p* < 0.0001) and to have received both pre-test (78.0% vs. 33.7%, *p* < 0.0001) and post-test counseling (80.9% vs. 60.5%, *p* < 0.0001). There were no differences in reported receipt of results or sharing of results. Within populations (encompassing intervention and comparison groups), testing ranged from 50.4% among MSM to 64.3% among FSW. Testing was highest among FSW sub-groups, for both the intervention (80.7 and 78.3% for SSW and KSW, respectively) and comparison (52.4 and 53.2%) groups. In addition, of those who were tested and received results, PWID participants reported the highest rates of HIV+ diagnoses across intervention and comparison groups (49.6 and 43.2%, respectively, difference not significant).

**Table 6:** HIV Testing Behaviors by Intervention vs. Comparison Group and Key Population

	Received pretest counseling n (%)				Tested for HIV n (%)				Of those who tested, received post-test counseling n (%)				Of those who tested, received results n (%)				Of those who tested and received results, HIV+ n (%)				Of those who tested HIV+, participant shared result with someone n (%)			
	Overall	Intervention	Comparison	*P* Value	Overall	Intervention	Comparison	*P* Value	Overall	Intervention	Comparison	*P* Value	Overall	Intervention	Comparison	*P* Value	Overall	Intervention	Comparison	*P* Value	Overall	Intervention	Comparison	*P* Value
ALL *n* = 2199	1228/2199 (55.8)	858/1100 (78.0)	370/1099 (33.7)	< 0.0001***	1354/2199 (61.6)	837/1100 (76.1)	517/1099 (47.0)	< 0.0001***	990/1354 (73.1)	677/837 (80.9)	313/517 (60.5)	< 0.0001***	1216/1354 (89.9)	750/837 (89.6)	466/517 (90.1)	0.75	236/1216 (19.4)	164/750 (21.9)	72/466 (15.5)	0.02*	161/236 (68.2)	113/164 (68.9)	48/72 (66.7)	0.92
PWID *n* = 694	396/694 (57.1)	274/358 (76.5)	122/336 (36.3)	< 0.0001***	435/694 (62.7)	278/358 (77.6)	157/336 (46.7)	< 0.001***	316/435 (72.6)	221/278 (79.5)	95/157 (60.5)	< 0.001***	376/435 (86.4)	244/278 (87.8)	132/157 (84.1)	0.42	178/376 (47.3)	121/244 (49.6)	57/132 (43.2)	0.43	121/178 (68)	82/121 (67.8)	39/57 (68.4)	0.92
MSM *n* = 337	169/337 (50.1)	116/161 (72.0)	53/176 (30.1)	< 0.0001***	170/337 (50.4)	109/161 (67.7)	61/176 (34.7)	< 0.001***	119/170 (70.0)	83/109 (76.1)	36/61 (59.0)	0.04*	148/170 (87.1)	93/109 (85.3)	55/61 (90.2)	0.56	9/148 (6.1)	6/93 (6.5)	3/55 (5.5)	0.80	5/9 (55.6)	3/6 (50.0)	2/3 (66.7)	0.64
CSW *n* =1367	767/1367 (56.1)	539/680 (79.3)	228/687 (33.2)	< 0.0001***	879/1367 (64.3)	535/680 (78.7)	344/687 (50.1)	< 0.001***	640/879 (72.8)	430/535 (80.4)	210/344 (61.0)	< 0.001***	803/879 (91.4)	485/535 (90.7)	318/344 (92.4)	0.67	89/803 (11.1)	63/485 (13.0)	26/318 (8.2)	0.07	59/89 (66.3)	42/63 (66.7)	17/26 (65.4)	0.82
CSW sub-groups																								
SSW *n* = 594	346/594 (58.2)	243/300 (81.0)	103/294 (35.0)	< 0.0001***	396/594 (66.7)	242/300 (80.7)	154/294 (52.4)	< 0.0001***	296/396 (74.7)	195/242 (80.6)	101/154 (65.6)	0.004**	360/396 (90.9)	217/242 (89.7)	143/154 (92.9)	0.31	46/360 (12.8)	28/217 (12.9)	18/143 (12.6)	0.93	29/46 (63)	16/28 (57.1)	13/18 (72.2)	0.49
KSW *n* = 599	342/599 (57.1)	242/300 (80.7)	100/299 (33.4)	< 0.0001***	394/599 (65.8)	235/300 (78.3)	159/299 (53.2)	< 0.0001***	283/394 (71.8)	192/235 (81.7)	91/159 (57.2)	< 0.0001***	364/394 (92.4)	216/235 (91.9)	148/159 (93.1)	0.50	30/364 (8.2)	25/216 (11.6)	5/148 (3.4)	0.01*	24/30 (80)	20/25 (80.0)	4/5 (80.0)	0.99

*Significant at *p* < 0.05; **Significant at *p* < 0.01; ***Significant at *p* < 0.001

The qualitative data underscored both the impact of outreach efforts on encouraging HIV testing, and persistent barriers in reaching key populations with testing services; attitudes towards health workers revealed a strong sense of continued stigmatization of individuals suspected of engaging in sex work, gay sex, and drug use (see Table 3). Several IDI participants admitted continued fear of learning their status. One PWID in Ha Noi noted that sometimes he lied to the outreach workers, saying that he had been tested when that was not true; he remained too fearful to be tested.

## Discussion

This mixed methods evaluation provides important evidence of the value of community outreach interventions deploying peers and other health educators for improving knowledge of HIV transmission, prevention, and treatment. Community members who interacted with peer and health educators demonstrated significantly greater HIV/AIDS knowledge, particularly on treatment questions, than those who did not. Intervention participants were more likely to have been tested for HIV and to have received counselling before and after testing. Likewise, outreach programs appear to have reduced risky sexual behaviors and increased uptake of HIV testing services among high-risk populations. As Vietnam and development partners plan funding and programmatic priorities to meet the 90-90-90 targets by 2020 and to end new infections by 2030, gaps in treatment knowledge, HIV testing, and consistent condom use demand sustained focus and funding. Our 2007–2008 evaluation data, combined with other recent research, offer critical guidance for national strategy and future program implementation.

### Sustained treatment & adherence

While intervention participants had higher levels of treatment knowledge than comparison participants in this study, treatment knowledge was still low among those receiving outreach. This is consistent with studies in Vietnam that have found numerous misconceptions regarding treatment—including one study which found that most respondents (70%) incorrectly believed that ART could cure HIV [24]. At the time of this evaluation, outreach workers were not regularly giving treatment information to clients, which may explain the low scores on HIV treatment knowledge questions among those receiving outreach [11]. However, recent research suggests that this gap in treatment knowledge persists [17, 24]. Looking forward, education about both the benefits and management of side effects of ART must remain a top priority. All stakeholders must remain vigilant about dispelling misconceptions about treatment, which negatively affect patients’ engagement, adherence to treatment, and clinical outcomes [25, 26].

### Engagement in HIV counseling and testing services

On the topic of HCT, we found that the intervention group was significantly more likely to receive voluntary pre- and post-test counseling, and HIV testing than the comparison group. These findings suggest that community outreach is a valuable strategy for increasing uptake of HIV testing services across key populations. This observation is consistent with findings from another study in Vietnam, which found that receiving voluntary HIV testing services, condoms, and injection equipment from the local HIV prevention program was significantly associated with lower reported HIV risk behavior [27]. Previous research has also found that HCT is an effective prevention strategy, and can be an entry point to support and care for key populations [28–30]. Nonetheless, key populations may still be reluctant to seek HIV testing even though they are aware of its importance [31]. We documented a number of reasons for this, including judgmental attitudes of clinical staff, fear of learning of one’s status, failure to prioritize testing given busy lives and perceived good health, and cost of related services.

### Getting to zero

Interventions focused on improving consistent condom use with all partners will also need to remain a high priority looking forward to the 2030 goal of ending AIDS. Although we found strong evidence of effectiveness of outreach in the higher condom use of intervention participants vis-à-vis comparison participants, over half of intervention participants reported inconsistent condom use, except with sex work clients. This is worrisome given more recent evidence that condom use has remained low in Vietnam [32, 33]. Assessments from India are informative, indicating similar patterns of low condom use among regular partners, and by men when having sex with women [34, 35]. This suggests that condom negotiation with repeat clients and regular partners is more difficult than with casual/new partners, and that this complexity may not be addressed easily via community-outreach programs [35]. Other approaches—such as empowerment, increased self-esteem, and community mobilization—may have a role in increasing condom use among regular sexual partners, and might be considered when developing interventions [36]. Strategies shown to be successful in increasing condom use with regular partners include providing high-intensity peer and clinical services for high-risk MSM and transgender people, and increasing condom availability among key populations [35]. Given rising concern about HIV among MSM, reaching this subpopulation will be critical [37]. Outreach efforts that make use of social media with MSM in other settings, including in sub-Saharan Africa, may hold lessons for places like Vietnam that are highly “wired,” with broad internet availability and mobile phone use [38–40]. Regardless of specific approach, prevention programs need to stress the importance of consistent condom use with all partners, regardless of sex, sexual orientation, or gender identity.

### Limitations

Several study limitations may bear on these findings. First, while the mixed methods approach allowed for triangulation of findings, the survey was cross-sectional, and thus cause and effect cannot be confirmed. Second, because sampling was respondent-driven rather than randomized, our results may be biased due the socio-demographic composition of either or both key population groups (intervention or comparison). In particular, reliance on social networks to recruit participants may have produced a sample with similar characteristics and similar behavioral tendencies, thus leading to skewed results [41]. Third, due to the self-reporting nature of the study, social desirability may have influenced our findings, as participants may have answered questions based on what they thought interviewers wanted to hear. Fourth, despite its breadth, our sample may not be representative of Vietnam’s at-risk population nationally since no sampling frame exists that lists these individuals by behavioral characteristics. Fifth, it is conceivable that participants had benefited from other interventions beyond the peer-outreach programs we assessed. However, to our knowledge, there were no other similar programs operating in Vietnam at that time. There may have been small programs distributing condoms in some of these places, but the individuals who participated in our study were all engaged in one or more illegal and heavily-stigmatized behaviors that meant they were a fairly hidden population and receiving HIV-related information and support primarily through the outreach programs. Moreover, were there other programs, individuals in both groups would have accessed them, and thus they would not be the source of systematic bias.

Finally, the study was conducted over 10 years ago, and its relevance may be questioned. We would posit that the study had important strengths that warrant attention: a strong mixed-methods design with collection of rich, in-depth qualitative data; a creative design to yield robust comparisons between intervention and comparision group participants; a broad national scope; and inclusion of all three critical vulnerable populations in Vietnam (PWID, FSW, and MSM). To our knowledge, there is no published study with anything near the comprehensive scope of this evaluation in geographic, participant, and outcome data terms. Given that little has been published on HIV prevention outreach programs in Vietnam, where the epidemic continues to pose a major challenge and key populations continue to be highly vulnerable, we believe our findings may inform efforts to improve outcomes in the HIV care cascade in Vietnam and perhaps elsewhere in Asia.

## Conclusions

This national evaluation led to important findings about the effectiveness of PEPFAR-supported community-based outreach programs targeting key populations of PWID, MSM, and FSW. The program appeared successful on numerous fronts, including increased HIV knowledge, high-risk injection and sexual behavior, and uptake of testing services. However, our evaluation identified important gaps which must be addressed in future prevention efforts, whether through large-scale community-outreach or through more intensive and clinic-based programming. Priorities should target low knowledge of HIV treatment among key populations, the complexity of sexual patterns and low condom use with regular partners, and inadequate awareness of and engagement with HCT services.

## Data Availability

The datasets generated and analysed during the current study are not publicly available currently because analyses of data by team members are still underway. Once these are completed, we will make data available from the corresponding author on reasonable request.

## Abbreviations

ART: Antiretroviral therapy
FGD: Focus group discussion
FSW: Female sex workers
HCMC: Ho Chi Minh City
HCT: HIV counseling and testing
IDI: In-depth interview
KSW: Female sex workers who work in establishments
MSM: Men who have sex with men
PEPFAR: President’s Emergency Plan for AIDS Relief
PWID: People who inject drugs
SSW: Female sex workers who work on the street
USG: United States Government
VND: Vietnamese Dong
